# An Experimental and Theoretical Study of the Effective Length of Embedded Scintillator Materials in End-Constructed Optical Fiber Radiation Sensing Probes

**DOI:** 10.3390/s25216704

**Published:** 2025-11-02

**Authors:** Yichen Li, Yong Feng, Jingjing Wang, Bo He, Ziyin Chen, Haojie Yang, Qieming Shi, Wenjing Hao, Jinqian Qian, Jiashun Luo, Jinhui Cui, Yongjun Liu, Tao Geng, Elfed Lewis, Weimin Sun

**Affiliations:** 1 Key Lab of In-Fiber Integrated Optics, Ministry Education of China, College of Physics and Optoelectronic Engineering, Harbin Engineering University, Harbin 150001, China; mool0114@163.com (Y.L.); wangjj@sjphotons.com (J.W.); _hebo@hrbeu.edu.cn (B.H.); _chenziyin@hrbeu.edu.cn (Z.C.); yhj15629563127@hrbeu.edu.cn (H.Y.); shiqieming@hrbeu.edu.cn (Q.S.); haowenjing@hrbeu.edu.cn (W.H.); qian-jq@neusoft.com (J.Q.); luojiashun@hrbeu.edu.cn (J.L.); cuijinhui@hrbeu.edu.cn (J.C.); liuyj@hrbeu.edu.cn (Y.L.); gengtao@hrbeu.edu.cn (T.G.); 2 Qingdao Huangdao District People’s Hospital, Qingdao 266400, China; fengyong@hrbeu.edu.cn; 3 First Affiliated Hospital of Harbin Medical University, Harbin 150001, China; 4 Optical Fiber Sensors Research Centre, University of Limerick, Castletroy, V94 T9PX Limerick, Ireland; elfed.lewis@ul.ie

**Keywords:** radiation detectors, fiber sensors, theoretical model, scintillators, effective length

## Abstract

**Highlights:**

**What are the main findings?**

- A method for constructing a simpler scintillator optical fiber radiation sensing probe is described.
- The efficiency of coupling scintillator luminescence into the optical fiber was analyzed, yielding the optimal length value for the scintillator sensing probe.

**What is the implication of the main finding?**

- To provide a base reference for sensor fabrication, thereby further enhancing the performance of fiber-optic radiation probes in terms of spatial resolution, signal-to-noise ratio, and other aspects.

**Abstract:**

Optical fiber radiation sensing probes made using inorganic scintillator materials have notable advantages in achieving high spatial resolution and building sensing arrays due to their small size and excellent linearity, serving as a key tool for dose measurement in precision radiotherapy. This study establishes a theoretical model for scintillator luminescence coupling into optical fibers, and derives a fluorescence intensity calculation formula based on the fiber’s numerical aperture and fluorescence self-absorption. The light intensity response to scintillator length for different absorption coefficients is established based on numerical simulation, providing a nonlinear fitting equation, resulting in a novel “effective length of scintillator” concept. Five probes with scintillator lengths of 0.2 mm, 0.5 mm, 1.0 mm, 1.5 mm, and 2.0 mm were prepared in the laboratory using a 3:1 mass ratio mixture of UV-setting epoxy and Gd_2_O_2_S:Tb powder. Tests in a clinical radiation delivery setting showed good agreement between experimental data and theory, confirming optimum effective length of the scintillator as 0.62 mm. This study indicates that inorganic scintillators for end-constructed probes do need not need to be excessively long. Analyzing the effective length can reduce scintillator usage, simplify fabrication and processing, and enhance the probe’s spatial resolution without decreasing the signal-to-noise ratio, thus offering new insights for optimizing optical fiber radiation probes.

## 1. Introduction

Radiotherapy is a universally employed method for treating tumors [1,2]. To achieve truly ‘precision radiotherapy’, various radiation detection technologies have been developed [3,4,5]. Among these, optical fiber radiation probes have emerged as a highly effective detection method for precision radiotherapy due to their advantages of compact size, low optical loss, and excellent linearity [6,7,8].

Optical fiber radiation probes are typically formed by coupling optical fibers with scintillators. Four structural configurations have been employed: coated structures, embedded structures, end-constructed structures, and doped structures. (1) The coated structure was initially formulated during the early development phase of scintillator optical fiber dosimeters, employing coating technology to apply powdered scintillator onto the optical fiber. Fluorescence light propagation within the fiber adheres to the law of total internal reflection. Under this mechanism, only light incident at specific angles into the fiber end-face thus meeting the conditions for total internal reflection can propagate continuously within the core. Light entering the fiber from the side cannot generally be confined within the core for propagation [9,10]. (2) The embedded design structure requires drilling holes in the fiber core to be filled with scintillator material. This fiber probe potentially achieves the highest resolution, with structural dimensions reaching the micrometer level in the case of singlemode glass. However, in practical applications, the brittleness of quartz material and stringent requirements for processing precision necessitate balancing dimensional accuracy with manufacturing feasibility. The drilling process faces dual challenges: excessive drilling depth compromises the fiber’s structural integrity, altering waveguide characteristics and thereby impairing the transmission efficiency and stability of received optical signals; conversely, insufficient drilling depth may result in inadequate scintillator filling resulting in inadequate sensitivity [11,12]. (3) The end-constructed structure was characterized by the scintillator being located on the end of the fiber, and the fluorescence generated light being coupled into the fiber. The latter type of sensor is relatively easily manufactured and has good Signal to Noise Ratio (SNR). This article aims to analyze the effective length of the scintillator from a theoretical and experimental perspective [13]. (4) The doping structure involves directly incorporating the scintillator during fiber fabrication, offering controllable probe diameter and repeatable operation. However, the manufacturing process is complex, with the scintillator undergoing high-temperature oxidation and hence exhibiting weak fluorescence signals [14,15,16]. Additionally, various sensors based on radiation-induced luminescence mechanisms were investigated in optical fibers [17,18]. Such non-scintillator-based sensing effectively avoids issues related to variable scintillator luminescence and fiber coupling efficiency. However, it remains challenging to obtain strong signals in low level radiation ranges. In the case of small-field radiation measurement, the size of individual fiber probes within integrated fiber sensing arrays significantly impacts the spatial accuracy of radiation dose measurement. Consequently, the miniaturization of single-point or array [19] optical fiber radiation probes represents considerable research value.

Taking the aforementioned structure into consideration, this article describes a method for constructing a cylindrical probe based on the above methods utilizing a mixture of UV-setting epoxy mixed with scintillators located at the end of a 0.98 mm diameter core plastic optical fiber (POF). This structure is relatively easy to fabricate and is lower in cost than using quartz optical fibers. To determine the effective length of the scintillator structure, a theoretical model was established describing the coupling of the scintillator luminescence into the fiber, alongside a formula for the fluorescence signal intensity generated within the fiber. This model provides an accurate reference for optimizing the size of the sensing probes of this study, thereby reducing the amount of scintillator material and probe volume whilst ensuring the signal strength required for use in clinical applications. Through simulation analysis, the relationship between the scintillator absorption coefficient and its effective construction length was ultimately derived. Finally, using hospital-based clinical radiotherapy delivered via a modern Linear Accelerator (Linac), the experimental test results of optical fiber radiation probes with scintillators of varying lengths constructed at the fiber end were compared with theory.

## 2. Scintillator-Fiber Coupling Model

**Figure 1:**
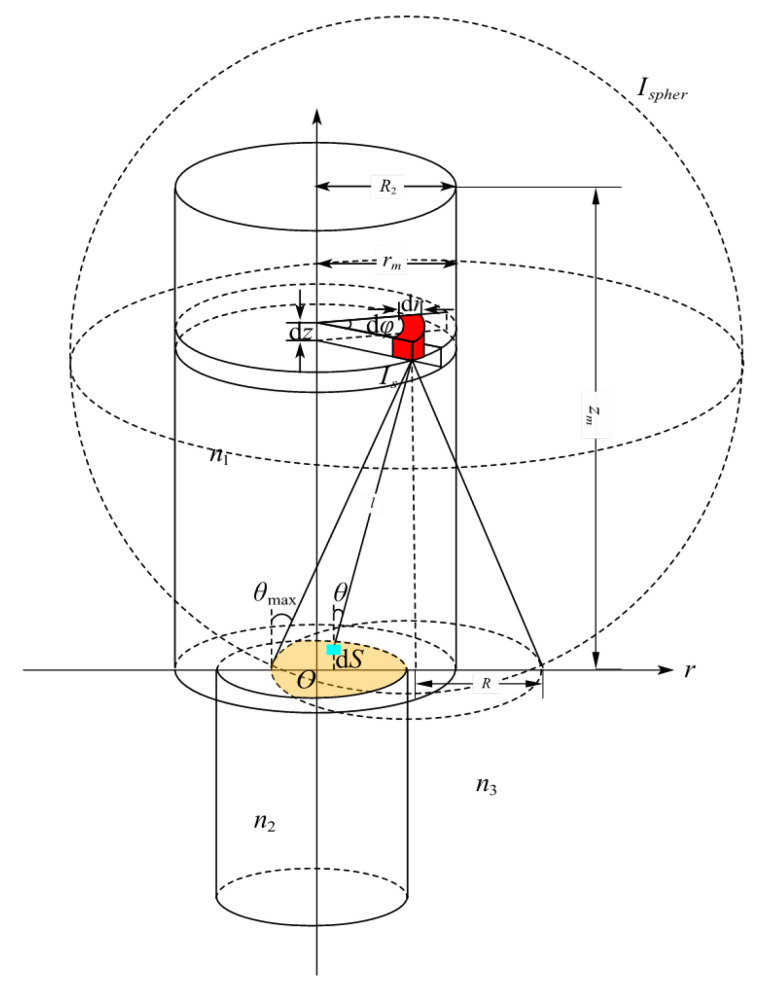
Model of scintillator luminescence coupled into an optical fiber.

A model was established to investigate the light collection efficiency of optical fiber radiation sensor probes defining light coupling from the scintillator material into the optical fiber, and it is shown in Figure 1. It is assumed that the scintillator is a light emitter with uniform density, and a volume element d*V* is taken from the scintillator, which corresponds to the red fan-shaped region in Figure 1. Therefore, the following holds:

(1) dV=rdφdrdz

(*r*, *φ*, *z*) (Figure 1) represent the coordinates of the volume element, d*V* of the scintillator in the cylindrical coordinate system, and its corresponding coordinates in the cartesian coordinate system are (*r*cos*φ*, *r*sin*φ*, *z*).

The intensity of the fluorescent light emitted by this volume element d*V* in the radiation field is proportional to its volume; therefore, its light intensity may be expressed as follows:

(2) $$dI_{s}=Ard\varphi drdz$$

*A* denotes the luminous intensity of the scintillator per unit volume under X-ray irradiation.

Assuming d*V* is a point source emitting spherical waves in all directions, for the fluorescence intensity emitted by the point source to be collected by the optical fiber and propagate within it in a guided-mode form, the angle of incidence of the rays entering the fiber must be less than the fiber’s maximum aperture angle *θ_max_*, i.e., only light within the cone depicted in Figure 1 satisfies the fiber’s guided-mode condition. The numerical aperture (*N.A.*) of the optical fiber is:

(3) $$NA=n_{1}sin\theta_{max}=\sqrt{n_{2}^{2}-n_{3}^{2}}$$

n_1_ denotes the refractive index of the scintillator, while n_2_ and n_3_ represent the refractive indices of the fiber core and cladding, respectively. *θ*_max_ is the maximum angle of light emission within the scintillator.

**Figure 2:**
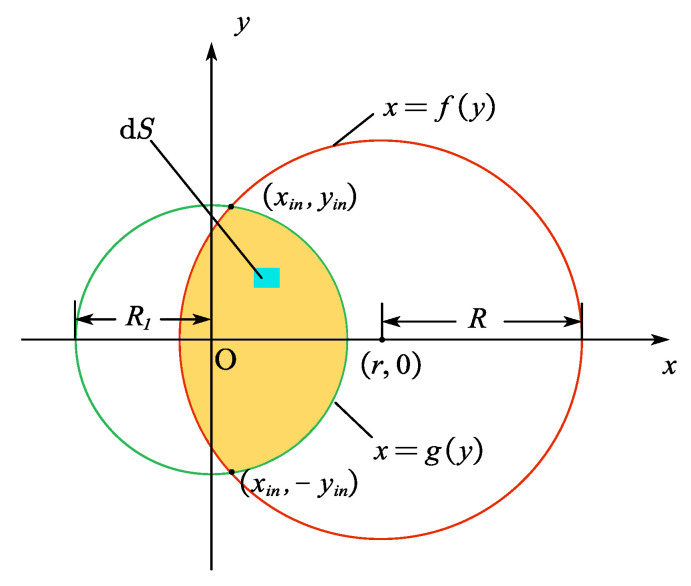
Schematic diagram of effective coupling area.

Only the overlapping region (yellow area in Figure 1 and Figure 2) between the conical projection’s circular zone on the fiber end-face (red circle in Figure 2, defined by *x* = *f* (*y*)) and the fiber core (green circle in Figure 2, defined by *x* = *g*(*y*)) enables successful light coupling into the core for guided propagation. To obtain the total transmittable fluorescence intensity from the entire scintillator (upper cylinder in Figure 1), the emission was integrated from each volume element d*V* that satisfies the fiber’s guided-mode conditions. Additionally, fluorescence absorption by the scintillator segment adjacent to the fiber must be considered.

Consider an arbitrary surface element d*S* within the effective coupling region represented in Figure 2, located at (*x*, *y*, 0). The intensity of fluorescent signals emitted by the point source that couple into the fiber for propagation were determined considering the circular symmetry of the system and selecting the point source coordinates being (*r*, 0, *z*) as shown in Figure 1. From Equation (2), the emission intensity of the point source is d*I*_s_. Accounting for fluorescence absorption by the scintillator, the fluorescent intensity reaching d*S* from the point source can be expressed as follows:

(4) $$dI=dI_{s}\cdot e^{-\mathrm{\alpha L}}\cdot \frac{dS\cos\theta }{S_{sphere}}$$

α represents the attenuation coefficient of the Beer-Lambert law; $L=\sqrt{{\left(x-r\right)}^{2}+y^{2}+z^{2}}$ denotes the path length from the point source to the d*S* region; *S_sphere_* = 4*πL*^2^ denotes the surface area of the sphere centered at the point source with radius *L*; *θ* represents the angle between the path *L* and the *Z*-axis, where $cos\theta =\frac{z}{L}$ serves as the inclination factor of the projection plane. Dividing the solid angle dS corresponding to the region by 4*πL*^2^ yields the proportion of fluorescence emitted from the point source that reaches the yellow region. Integrating this calculation across the entire length of the scintillator provides the total intensity reaching this region from the point source.

(5) $$I_{fiber}=\int dI_{s}\int_{-y_{in}}^{y_{in}}dy\int_{f\left(y\right)}^{g\left(y\right)}dx\frac{e^{-\alpha L}z}{4\pi L^{3}}$$

*g*(*y*) and *f*(*y*) denote the boundaries of the effective illumination region. For the scenario in Figure 2, the upper and lower intersection points of the two circles *x* = *g*(*y*) and *x* = *f*(*y*) are (*x_in_*, *y_in_*,0) and (*x_in_*, −*y_in_*, 0), respectively. Owing to the vertical symmetry of the figure, the total guided light intensity can be obtained by calculating the intensity in the *y* > 0 half-region and multiplying it by 2. Substituting Equation (2) into Equation (5) yields the expression for the total intensity of light from the entire scintillator coupled into the fiber as follows:

(6) $$I_{fiber}=2A\int_{0}^{2\pi }d\varphi \int_{0}^{R_{2}}rdr\int_{0}^{z_{m}}dz\int_{0}^{y_{in}}dy\int_{f\left(y\right)}^{g\left(y\right)}dx\frac{z\cdot exp[-\alpha \sqrt{{\left(x-r\right)}^{2}+y^{2}+z^{2}}]}{4\pi {\left[{\left(x-r\right)}^{2}+y^{2}+z^{2}\right]}^{3/2}}$$

*z_m_* denotes the length of the scintillator, *R*_2_ represents the radius of the scintillator, and the arc function for the edge of the fiber core is:

(7) $$x=g(y)=\pm \sqrt{R_{1}^{2}-y^{2}}$$

while the arc function for the region of the effective conical projection is:

(8) $$x=f\left(y\right)=r\pm \sqrt{R^{2}-y^{2}}=r\pm \sqrt{z^{2}{tan}^{2}\theta_{max}-y^{2}}$$

**Figure 3:**
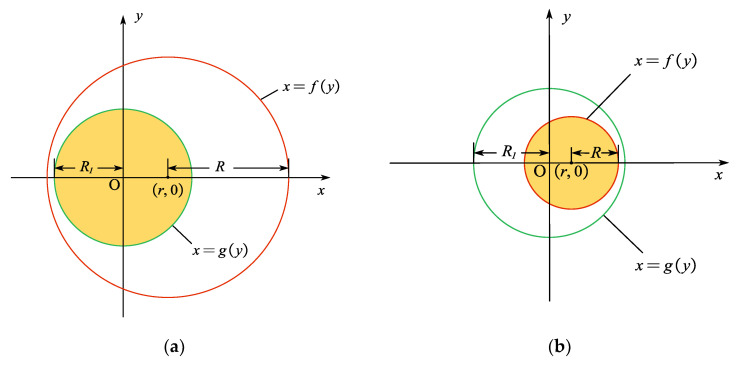

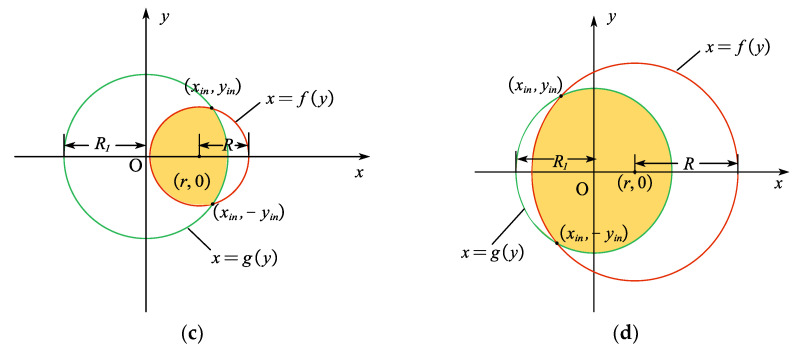
Several special coupling regions. (**a**) The scintillator luminous range (red) after point source emission fully encompasses the fiber end face (green) in the coordinate system; (**b**) The scintillator luminous range (red) after point source emission is fully encompassed by the fiber end face (green) in the coordinate system; (**c**) The scintillator luminous range (red) is a semi-major circle; (**d**) The fiber end face (green) in the coordinate system is a semi-major circle.

By solving Equations (7) and (8) simultaneously, the coordinates of the upper intersection points of the two circles can be obtained as follows:

(9) $$\left\{\begin{array}{c} x_{in}=\pm \sqrt{R_{1}^{2}-z^{2}{tan}^{2}\theta_{max}+\frac{{\left(z^{2}{tan}^{2}\theta_{max}+r^{2}-R_{1}^{2}\right)}^{2}}{4r^{2}}}\\ y_{in}=\sqrt{z^{2}{tan}^{2}\theta_{max}-\frac{{\left(z^{2}{tan}^{2}\theta_{max}+r^{2}-R_{1}^{2}\right)}^{2}}{4r^{2}}} \end{array}\right.$$

when the point source emits spherical waves, it can be categorized into six distinct cases based on the positional relationship between the light-emitting range of the scintillator and the fiber end-face. These cases are specified as follows:

(1) The light-emitting range of the point source (within the red circle) partially overlaps with the fiber end-face (within the green circle), with both circles corresponding to small semicircular arcs. Two scenarios arise: when *r* > *R*_1_ (point source projection point lies outside the core) and $r-R_{1}\leq R<\sqrt{R_{1}^{2}+r^{2}}$, or when *r* < *R*_1_ (point source projection point lies inside the core) and $\sqrt{R_{1}^{2}-r^{2}}\leq R<\sqrt{R_{1}^{2}+r^{2}}$, as illustrated in Figure 2. The transmitted light intensity in this case can be calculated using Formula (6).

(2) When the light-emitting range of the point source (within the red circle) fully encompasses the fiber end-face (within the green circle) within the coordinate system, 0 ≤ *r* ≤ *R* − *R*_1_, as shown in Figure 3a.

(10) $$I_{fiber}=2A\int_{0}^{2\pi }d\varphi \int_{0}^{R_{2}}rdr\int_{0}^{z_{m}}dz\int_{0}^{R_{1}}dy\int_{-\sqrt{R_{1}^{2}-y^{2}}}^{\sqrt{R_{1}^{2}-y^{2}}}dx\frac{z\cdot exp[-\alpha \sqrt{{\left(x-r\right)}^{2}+y^{2}+z^{2}}]}{4\pi {\left[{\left(x-r\right)}^{2}+y^{2}+z^{2}\right]}^{3/2}}$$

(3) When the light-emitting range of the point source (within the red circle) is entirely contained within the fiber end-face (within the green circle) in the coordinate system, 0 ≤ *r* ≤ *R*_1_ − *R*, as shown in Figure 3b.

(11) $$I_{fiber}=2A\int_{0}^{2\pi }d\varphi \int_{0}^{R_{2}}rdr\int_{0}^{z_{m}}dz\int_{0}^{R}dy\int_{r-\sqrt{R^{2}-y^{2}}}^{r+\sqrt{R^{2}-y^{2}}}dx\frac{z\cdot exp[-\alpha \sqrt{{\left(x-r\right)}^{2}+y^{2}+z^{2}}]}{4\pi {\left[{\left(x-r\right)}^{2}+y^{2}+z^{2}\right]}^{3/2}}$$

(4) Where the red circle forms a large semicircle, then $0<R_{1}-r<R\leq \sqrt{R_{1}^{2}-r^{2}}$, as shown in Figure 3c. In this case, an additional region must be integrated above the *y_in_*.

(12) $$I_{fiber}=2A\int_{0}^{2\pi }d\varphi \int_{0}^{R_{2}}rdr\int_{0}^{z_{m}}dz\{\int_{0}^{y_{in}}dy\int_{f\left(y\right)}^{g\left(y\right)}dx\frac{z\cdot exp[-\alpha \sqrt{{\left(x-r\right)}^{2}+y^{2}+z^{2}}]}{4\pi {\left[{\left(x-r\right)}^{2}+y^{2}+z^{2}\right]}^{3/2}}\;+\int_{y_{in}}^{R}dy\int_{r-\sqrt{R^{2}-y^{2}}}^{r+\sqrt{R^{2}-y^{2}}}dx\frac{z\cdot exp[-\alpha \sqrt{{\left(x-r\right)}^{2}+y^{2}+z^{2}}]}{4\pi {\left[{\left(x-r\right)}^{2}+y^{2}+z^{2}\right]}^{3/2}}\}$$

(5) Where the green circle forms a large semicircle, then $\sqrt{R_{1}^{2}+r^{2}}\leq R<R_{1}+r$, as shown in Figure 3d. In this case, an additional region must be integrated above the *y_in_*.

(13) $$I_{fiber}=2A\int_{0}^{2\pi }d\varphi \int_{0}^{R_{2}}rdr\int_{0}^{z_{m}}dz\{\int_{0}^{y_{in}}dy\int_{f\left(y\right)}^{g\left(y\right)}dx\frac{z\cdot exp[-\alpha \sqrt{{\left(x-r\right)}^{2}+y^{2}+z^{2}}]}{4\pi {\left[{\left(x-r\right)}^{2}+y^{2}+z^{2}\right]}^{3/2}}\;+\int_{y_{in}}^{R_{1}}dy\int_{-\sqrt{R_{1}^{2}-y^{2}}}^{\sqrt{R_{1}^{2}-y^{2}}}dx\frac{z\cdot exp[-\alpha \sqrt{{\left(x-r\right)}^{2}+y^{2}+z^{2}}]}{4\pi {\left[{\left(x-r\right)}^{2}+y^{2}+z^{2}\right]}^{3/2}}\}$$

(6) Where the luminescent range of the point source after emission does not intersect with the fiber end-face in the coordinate system, then *r* > *R* + *R*1, *I_fiber_* = 0.

Based on this scintillator-fiber coupling model, the fluorescent intensity received by the fiber from the point source at different locations can be calculated using Equation (6) and Equations (10)–(13), according to the different positional relationships between the red circle and the green circle. Subsequently, integration is performed over the guided fluorescent intensity emitted by the entire scintillator.

## 3. Simulation Design

To validate the theoretical model and evaluate the optical performance of the designed probe, several numerical simulations were conducted to analyze the variation in scintillator length under different absorption conditions. This led to the introduction of the concept of effective length as a reference for determining the construction length of the scintillator.

### 3.1. Selection of Scintillator Materials and Absorption Coefficient α

The absorption coefficient (*α*) is a physical parameter that characterizes a medium’s ability to absorb light at specific wavelengths, and its value is dependent on both the properties of the medium and the wavelength of the incident light. Notably, *α* has a significant impact on the fluorescence intensity detected using optical fibers.

**Figure 4:**
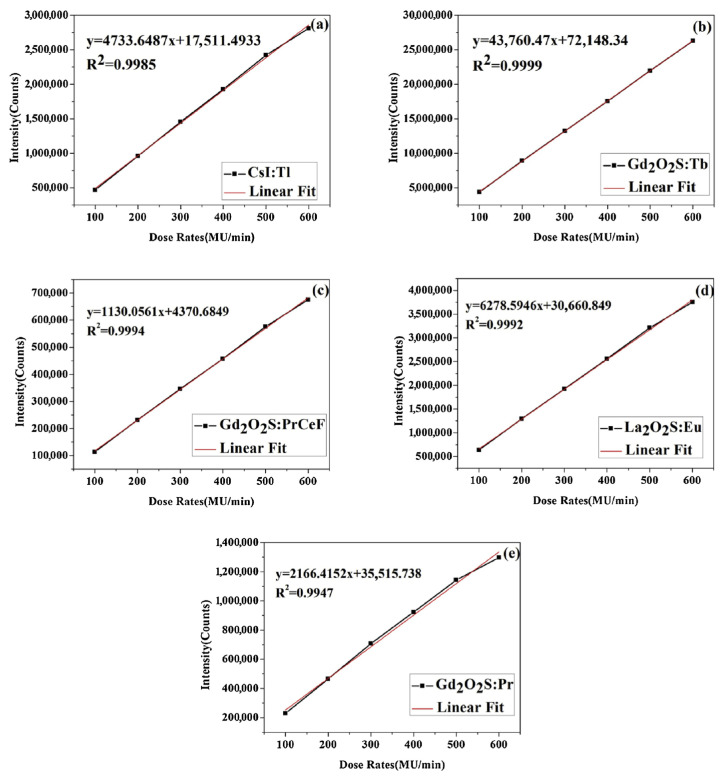
The linearity of the output intensity with varying dose rates. (**a**) sample 1 used CsI:Tl as scintillator material; (**b**) sample 2 used Gd_2_O_2_S:Tb as scintillator material; (**c**) sample 3 used Gd_2_O_2_S:PrCeF as scintillator material; (**d**) sample 4 used La_2_O_2_S:Eu as scintillator material; (**e**) sample 5 used Gd_2_O_2_S:Pr as scintillator material [20].

Several scintillator materials have previously investigated by the authors of this article, namely CsI:Tl, Gd_2_O_2_S:Pr, Ce, F, Gd_2_O_2_S:Tb, La_2_O_2_S:Eu and Gd_2_O_2_S:Pr. Experiments to investigate the linearity of the dosimeters with different scintillator materials were conducted over six exposures by increasing the dose rates. The results of the experiments described above demonstrate that La_2_O_2_S:Eu and Gd_2_O_2_S:Tb are both highly attractive materials for use in optical fiber dosimeters in terms of output intensity and resulting SNR compared to other materials investigated. Gd_2_O_2_S:Tb has greater stability as the dose rate increases compared to the other four scintillator materials shown in Figure 4 [20,21].

Through a comprehensive evaluation of their material properties, the inorganic scintillator Terbium activated Gadolinium Oxy-Sulfide (Gd_2_O_2_S:Tb, UKL65/UF-R1, Phosphor Technology, Kennesaw, GA, USA) was determined to exhibit the optimal fluorescence performance [22]. As a result, Gd_2_O_2_S:Tb was selected as the fluorescent material for the optical fiber radiation probe used in this study. Polymethyl methacrylate (PMMA, ESKA CH4001, Mitsubishi Corporation, Chiyoda-ku, Tokyo, Japan) fiber was selected as the optical transmission fiber, whose operating wavelength range is well matched to the central wavelength of 545 nm of the fluorescence signal emitted by Gd_2_O_2_S:Tb [11].

To investigate the relationship between received light intensity and scintillator construction length, representative absorption coefficient values of *α* = 0.1 mm^−1^, 1 mm^−1^, and 10 mm^−1^ were selected. Based on these absorption coefficients and Equations (6) and (10)–(13), numerical simulations were employed to plot the variation curve of fiber received light intensity *I_fiber_* with scintillator construction length *z_m_*.

### 3.2. Simulation Results

**Figure 5:**
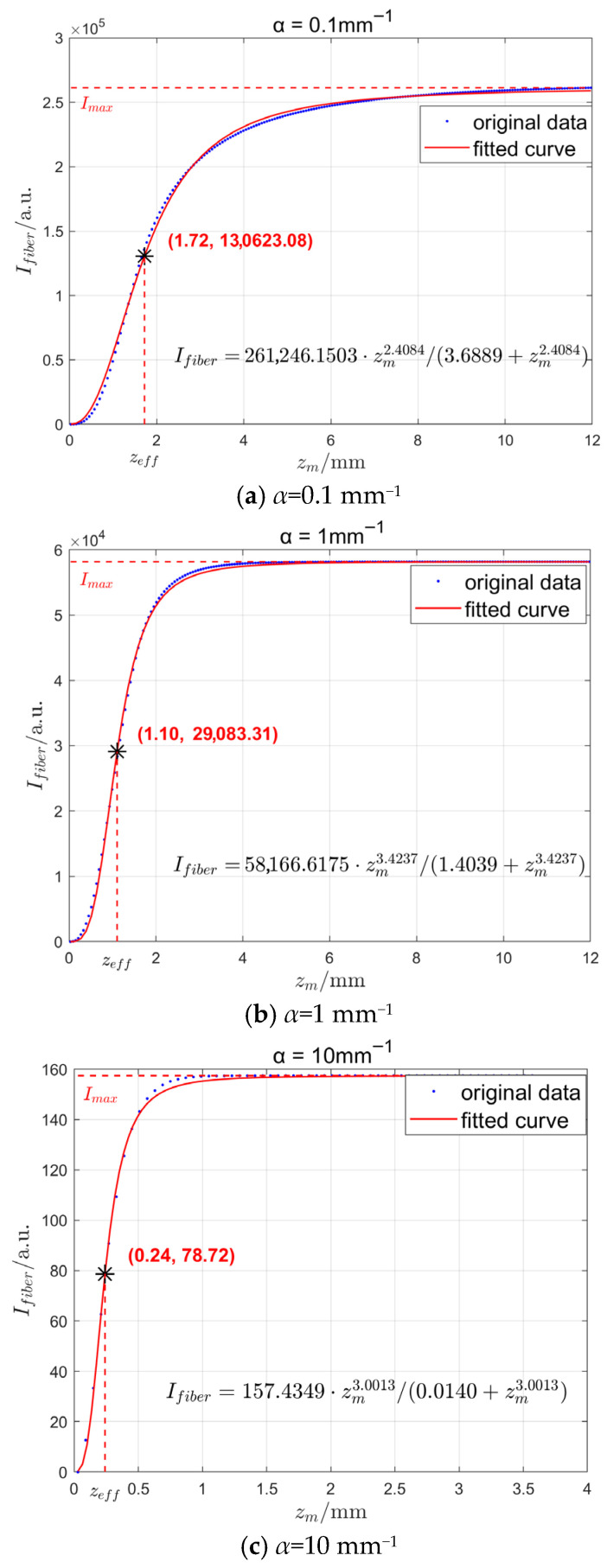
The simulated intensity of the fluorescence signal guided by the PMMA fiber with different absorption coefficients.

In the fabrication of the optical fiber radiation probe, a PMMA optical fiber with a numerical aperture (*N.A.*) of 0.5 and a core radius (*R*_1_) of 0.49 mm was selected, which features low attenuation characteristics in the visible wavelength band, coupled with low cost and durability. The radius of the scintillator (*R*_2_) was set to 0.5 mm. The simulation results under the conditions with absorption coefficient (*α*) values of 0.1 mm^−1^, 1 mm^−1^, and 10 mm^−1^ are presented in Figure 5.

As shown in Figure 5, the relationship function between the transmitted fluorescence intensity *I_fiber_* of the scintillator and *z_m_* has an asymptote, and the scintillator length corresponding to the intensity reaching its extreme value is related to the absorption coefficient α. As *z_m_* increases, the received intensity *I_fiber_* approaches a limiting value. This analysis method used in this paper defines the effective length *z_eff_* as the scintillator length reaching half this limit value. Consequently, when fabricating fiber probes, a length slightly exceeding the effective length suffices to achieve substantial fluorescence intensity whilst enhancing the spatial resolution of radiation field detection. The blue discrete points in Figure 5 represent the increment in scintillator length *z_m_* during simulation. The numerical calculation of light intensity received at the fiber end-face was performed using the integration model described in the preceding section. Based on the computational results, a function capable of accurately fitting the data was selected. The red curve depicted in Figure 5 represents this fitted curve.

Nonlinear fitting of the simulation data from Figure 5 yielded the following fitting function:

(14) $$I_{fiber}=\frac{I_{max}z_{m}^{P}}{z_{m}^{P}+q}$$

*I_max_* represents the curve’s maximum value, which is a function of both parameters *p* and *q* as well as the abs. *p* and *q* are parameters automatically fitted using a computer based numerical model on simulation data.

In the case of the fluorescence detection, the half-value point is frequently employed as a characteristic parameter for signal attenuation. To ensure the signal-to-noise ratio meets detection requirements, the scintillator length was selected at which the light intensity reduces to half its maximum value as the effective length of the probe. From Equation (14), when *I_fiber_* = *I_max_*/2, the effective length is obtained as

(15) $$z_{eff}=\sqrt[{p}]{q}$$

**Figure 6:**
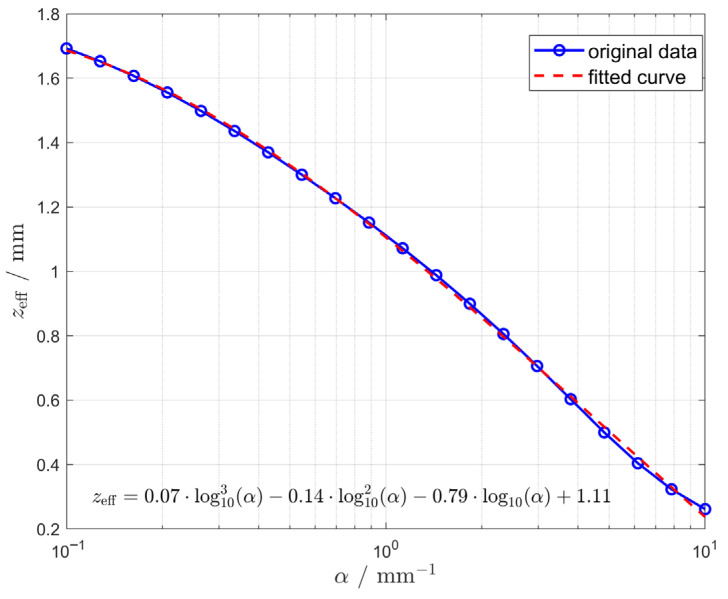
Curve fitting results of the relationship between absorption coefficients and the effective lengths of scintillator.

The simulation further calculated the effective length for 20 data points of the absorption coefficient *α* ranging from 0.1 mm^−1^ to 10 mm^−1^, and fitted these points in a coordinate system with the horizontal axis on a logarithmic scale. The blue dots in Figure 6 represent the actual calculated values of effective length at different alpha values, while the red curve depicts the fitted curve selected to most closely approximate the trend in the calculated results.

It should be noted that the effective length calculated serves solely as a theoretical design reference, not the actual precise length of the sensor element. The analysis indicates that the probe may be deployed provided the signal strength reaches the required magnitude. However, accurate calibration of each sensor remains essential during actual fabrication, testing, and usage.

## 4. Experimental Verification

To validate the simulation results, experimental sensor probe structures were fabricated with scintillator lengths of 0.2 mm, 0.5 mm, 1.0 mm, 1.5 mm, and 2.0 mm located at the end faces of five separate but identical optical fibers and bonded using ultraviolet light epoxy curing. The experimental relationship between the effective scintillator length and the fluorescence intensity transmitted through the fiber was therefore studied.

### 4.1. Fabrication of Optical Fiber Probes and Establishment of Experimental Facilities

**Figure 7:**
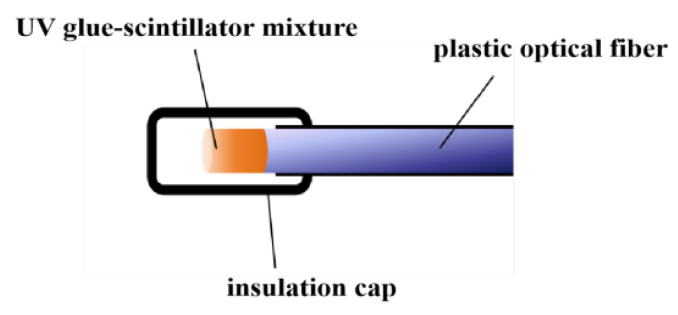
Schematic structure of a PMMA optical fiber X-ray sensing probe.

This experiment primarily used a 10 m length PMMA optical fiber with a core diameter of 0.98 mm. On one end of this fiber, a mixture of UV-setting epoxy (D-5608, Zhuolide, Foshan, China) and Gd_2_O_2_S:Tb powder was constructed by blending at a 3:1 mass ratio and subsequently cured under exposure to ultraviolet light. The mixture was uniformly applied to the end face of the plastic optical fiber through successive coating steps. Following each coating application, the UV adhesive underwent photopolymerisation under ultraviolet light, firmly anchoring the Gd_2_O_2_S:Tb powder to the fiber end-face. This coating process was repeated until the scintillator had grown to a predetermined length on the fiber end-face, thereby completing the fabrication of the optical fiber radiation probe. The entire PMMA optical fiber radiation probe structure is illustrated in Figure 7.

**Figure 8:**
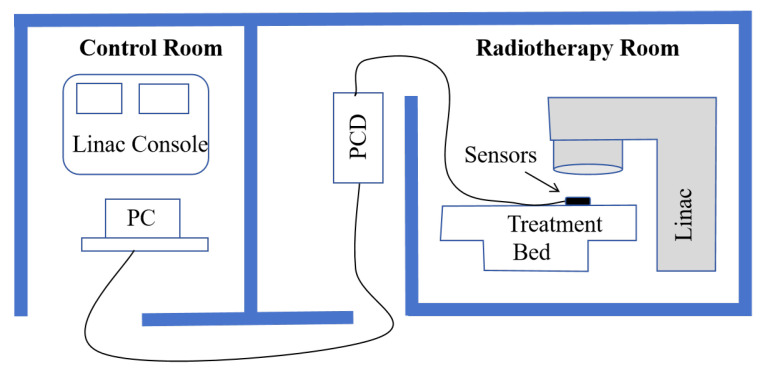
Schematic Diagram of the Clinical Experimental Environment.

The experimental testing was conducted at the Department of Radiation Oncology at the First Affiliated Hospital of Harbin Medical University. A Varian IX-3937 linear accelerator(Varian Medical Systems, Palo Alto, California, CA, USA) was used as the radiation source, generating a 6 MV X-ray beam. Experiments were conducted at a tightly controlled environmental room temperature (20 °C). The schematic of the clinical experimental environment is shown in Figure 8.

**Figure 9:**
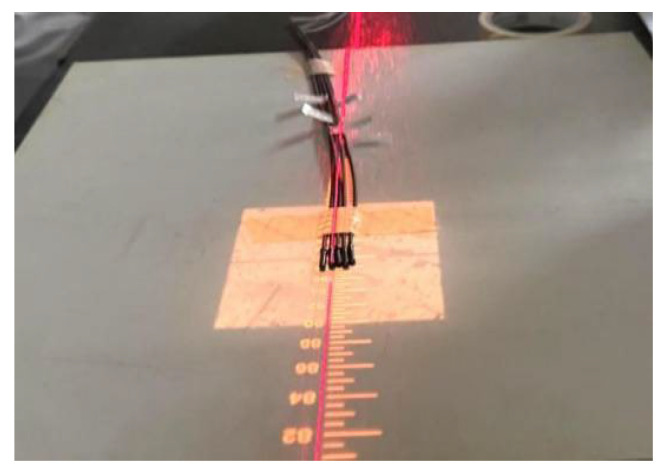
Probes at the radiotherapy field center on a treatment bed.

Five optical fiber probes were fixed on the treatment bed in the radiotherapy room, positioned at the center of the 10 × 10 cm^2^ treatment field. The beam delivery head of the linear accelerator was located directly above and centered with the optical fiber probes, as illustrated in Figure 9. The distal ends of the five optical fiber probes were connected to five separate channels of a photon-counting detector (PCD, Model CH326, Hamamatsu, Hamamatsu City, Japan). PCD converts were positioned 10 m from the radiation source and behind a shielding wall. The received light signals were converted into digital output signals (counts). These digital signals are transmitted via USB cables to a PC in the control room for data acquisition.

### 4.2. Experimental Test Results

Performance testing of the probe was conducted using 6 MV energy X-ray conditions. During the experiment, five optical fiber radiation probes with varying lengths of scintillator filling were sequentially tested at different dose rates. According to the 2016 standard “Outline for the Evaluation of Treatment-Level Ionization Chamber Dosimeters” [23] stipulates that in dose rates nonlinearity testing, “nonlinearity shall be measured at five equidistant points within each decade of the measurement range.” Therefore, in conventional radiotherapy, dose rates of 100, 200, 300, 400, 500, and 600 cGy/min are commonly used parameter settings. This paper tests the linearity of optical fiber probe dose rates based on actual radiotherapy applications, employing six sampling points within this dose rate range.

**Figure 10:**
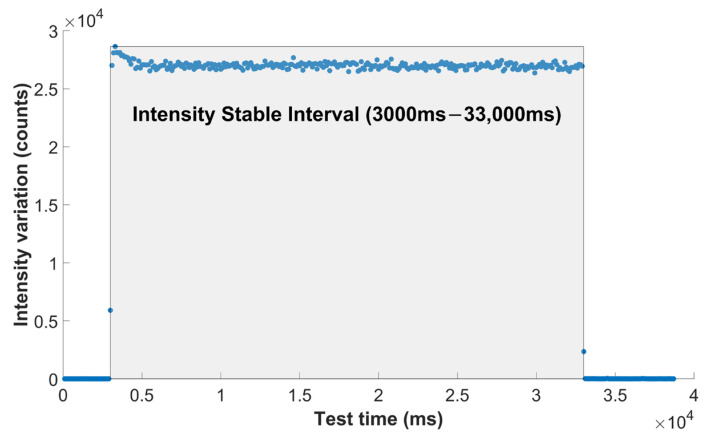
The changing intensity of the 0.5 mm-length probe at 400 cGy/min with time.

In the performance testing scenario of this optical fiber X-ray sensor, the average intensity value was used to evaluate the X-ray response of five probe prototypes with different scintillator lengths. The average intensity refers to the mean optical signal intensity received by the sensor per unit time under irradiation at a specific dose rate. Taking the data of the 0.5 mm-length probe with a dose rate of 400 cGy/min as an example (Figure 10), for the calculation of average light intensity, the time interval where the PCD’s light intensity response stabilized (3000 ms~33,000 ms) was chosen.

**Figure 11:**
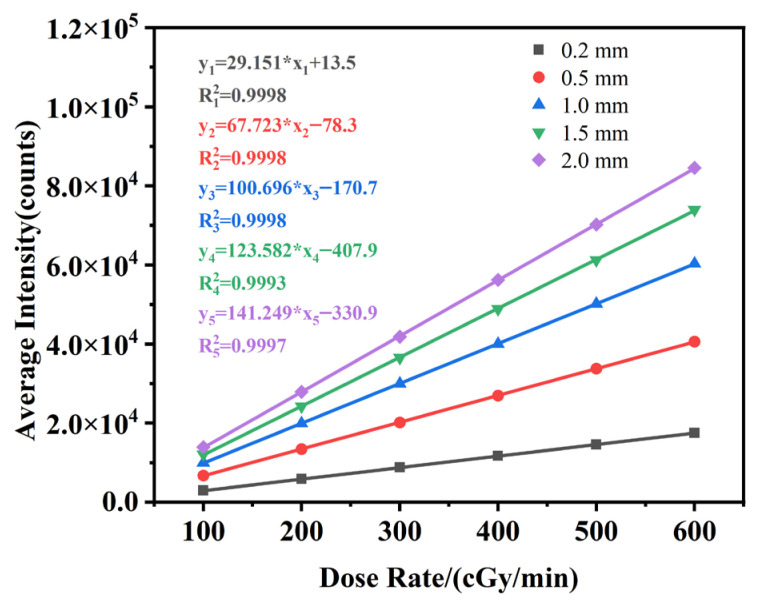
The average light intensity of optical fiber radiation probes with different scintillators filling lengths at various dose rates.

The average optical intensity output from the five optical fibers under the irradiation conditions with a dose rate ranging from 100 to 600 cGy/min are plotted in Figure 11.

In the equation fitted to this graph, *x_i_* represents the radiation dose rate received by the independent variable optical fiber probe cable, while *y_i_* represents the number of photons detected by the detector. The *i* value ranges in integer values from 1 to 5 to represent probes of different lengths from 0.2 to 2.0 mm. The results demonstrate that the scintillator in the optical fiber radiation probe exhibits an excellent linear response to X-ray dose rate. The linear fit coefficient R^2^ for the dose rates are better than 0.999 in each case, demonstrating beneficial sensing characteristics.

**Figure 12:**
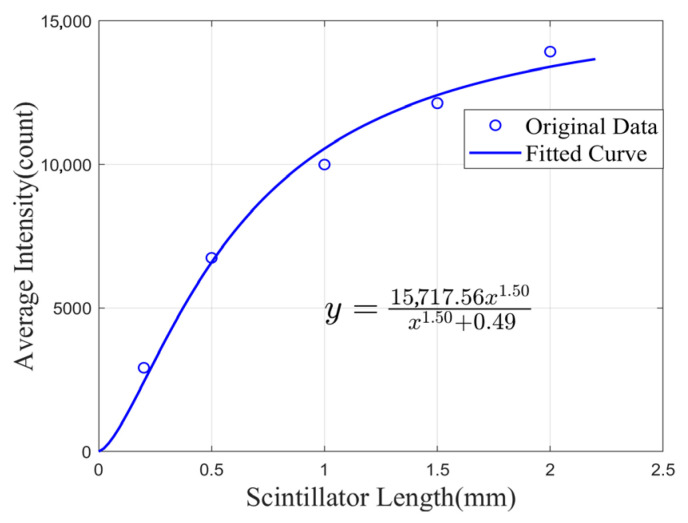
Fitting curves of the received light intensity as a function of the scintillator length under the same radiation dose.

Under the condition of identical irradiation dose, the optical intensity shows a gradually decelerating trend with the increase in scintillator length. This phenomenon has been discussed in depth in the model construction section. The measured results of scintillator construction lengths and their corresponding optical intensities were fitted using Equation (14), as shown in Figure 12.

In Figure 12, the independent variable “*x*” corresponds to the scintillator length, while the dependent variable “*y*” corresponds to the number of photons received by the detector. With $R^{2}>0.99$ for the fitting equation, and parameters *p* = 1.5 and *q* = 0.49, *z_eff_* = 0.62 mm was calculated. This result indicates that the proposed theoretical model can provide a scientific reference for the fabrication of practical probes. When fabricating the probe, referencing this length prevents excessive attenuation of the optical intensity signal (reduced by several orders of magnitude), which would otherwise compromise the signal-to-noise ratio. It also enhances the spatial resolution for single-point detection while conserving costly materials.

## 5. Conclusions

In conclusion, Gd_2_O_2_S:Tb scintillators exhibit excellent linearity when applied to clinical radiotherapy X-ray dose sensing. Nevertheless, they suffer from intrinsic fluorescence self-absorption: as the length of the scintillator probe reaches a certain threshold, the fluorescent photons emitted from the distal end fail to couple into the optical fiber, leading to saturation of the signal intensity measured using the PCD (detector). Based on this phenomenon, the present study has successfully proposed the concept of “effective length” through theoretical modeling and numerical simulations. The scintillator probe was fabricated by mixing UV-setting epoxy and Gd_2_O_2_S:Tb scintillator powder at a mass ratio of 3:1. Additionally, the proposed End-Constructed process facilitated the fabrication of optical fiber radiation sensors with scintillators of varying lengths, offering superior consistency and easier length control compared to the previously reported embedded structure. Clinical experimental results have demonstrated an excellent linear fit (R^2^ > 0.99) with the theoretical simulation, validating the rationality of the proposed model. The calculated effective length of the mixed-material sensor element in this case was 0.62 mm.

This effective length serves as a useful reference value. If key parameters of the scintillator were changed, e.g., refractive index, absorption coefficient, and doping concentration, the proposed method enables the determination of an optimal probe length. Overall, this research provides a crucial reference for the miniaturized fabrication of single-point optical fiber radiation probes.

## Data Availability

The datasets generated during and analyzed during the current study are available from the corresponding author upon reasonable request.
